# Good practices for clinical data warehouse implementation: A case study in France

**DOI:** 10.1371/journal.pdig.0000298

**Published:** 2023-07-06

**Authors:** Matthieu Doutreligne, Adeline Degremont, Pierre-Alain Jachiet, Antoine Lamer, Xavier Tannier

**Affiliations:** 1 Mission Data, Haute Autorité de Santé, Saint-Denis, France; 2 Inria, Soda team, Palaiseau, France; 3 Univ. Lille, CHU Lille, ULR 2694—METRICS: Évaluation des Technologies de santé et des Pratiques médicales, Lille, France; 4 Fédération régionale de recherche en psychiatrie et santé mentale (F2RSM Psy), Hauts-de-France, Saint-André-Lez-Lille, France; 5 Sorbonne Université, Inserm, Université Sorbonne Paris-Nord, Laboratoire d’informatique médicale et d’ingénierie des connaissances en e-Santé, LIMICS, France; Yonsei University College of Medicine, REPUBLIC OF KOREA

## Abstract

Real-world data (RWD) bears great promises to improve the quality of care. However, specific infrastructures and methodologies are required to derive robust knowledge and brings innovations to the patient. Drawing upon the national case study of the 32 French regional and university hospitals governance, we highlight key aspects of modern clinical data warehouses (CDWs): governance, transparency, types of data, data reuse, technical tools, documentation, and data quality control processes. Semi-structured interviews as well as a review of reported studies on French CDWs were conducted in a semi-structured manner from March to November 2022. Out of 32 regional and university hospitals in France, 14 have a CDW in production, 5 are experimenting, 5 have a prospective CDW project, 8 did not have any CDW project at the time of writing. The implementation of CDW in France dates from 2011 and accelerated in the late 2020. From this case study, we draw some general guidelines for CDWs. The actual orientation of CDWs towards research requires efforts in governance stabilization, standardization of data schema, and development in data quality and data documentation. Particular attention must be paid to the sustainability of the warehouse teams and to the multilevel governance. The transparency of the studies and the tools of transformation of the data must improve to allow successful multicentric data reuses as well as innovations in routine care.

## Author summary

Reusing routine care data does not come free of charges. Attention must be paid to the entire life cycle of the data to create robust knowledge and develop innovation. Building upon the first overview of CDWs in France, we document key aspects of the collection and organization of routine care data into homogeneous databases: governance, transparency, types of data, data reuse main objectives, technical tools, documentation, and data quality control processes. The landscape of CDWs in France dates from 2011 and accelerated in the late 2020, showing a progressive but still incomplete homogenization. National and European projects are emerging, supporting local initiatives in standardization, methodological work, and tooling. From this sample of CDWs, we draw general recommendations aimed at consolidating the potential of routine care data to improve healthcare. Particular attention must be paid to the sustainability of the warehouse teams and to the multilevel governance. The transparency of the data transformation tools and studies must improve to allow successful multicentric data reuses as well as innovations for the patient.

## Introduction

### Real-world data

Health information systems (HIS) are increasingly collecting routine care data [1–7]. This source of real-world data (RWD) [8] bears great promises to improve the quality of care. On the one hand, the use of this data translates into direct benefits—primary uses—for the patient by serving as the cornerstone of the developing personalized medicine [9,10]. They also bring indirect benefits—secondary uses—by accelerating and improving knowledge production: on pathologies [11], on the conditions of use of health products and technologies [12,13], on the measures of their safety [14], efficacy or usefulness in everyday practice [15]. They can also be used to assess the organizational impact of health products and technologies [16,17].

In recent years, health agencies in many countries have conducted extensive work to better support the generation and use of real-life data [8,17–19]. Study programs have been launched by regulatory agencies: the DARWIN EU program by the European Medicines Agency and the Real World Evidence Program by the Food and Drug Administration [20].

### Clinical data warehouse

In practice, the possibility of mobilizing these routinely collected data depends very much on their degree of concentration, in a gradient that goes from centralization in a single, homogenous HIS to fragmentation in a multitude of HIS with heterogeneous formats. The structure of the HIS reflects the governance structure. Thus, the ease of working with these data depends heavily on the organization of the healthcare actors. The 2 main sources of RWD are insurance claims—more centralized—and clinical data—more fragmented.

**Claims data** is often collected by national agencies into centralized repositories. In South Korea, the government agency responsible for healthcare system performance and quality (HIRA) is connected to the HIS of all healthcare stakeholders. HIRA data consists of national insurance claims [21]. England has a centralized healthcare system under the National Health Service (NHS). Despite not having detailed clinical data, this allowed the NHS to merge claims data with detailed data from 2 large urban medicine databases, corresponding to the 2 major software publishers [22]. This data is currently accessed through Opensafely, a first platform focused on Coronavirus Disease 2019 (COVID-19) research [23]. In the United States, even if scattered between different insurance providers, claims are pooled into large databases such as Medicare, Medicaid, or IBM MarketScan. Lastly, in Germany, the distinct federal claims have been centralized only very recently [24].

**Clinical data** on the other hand, tends to be distributed among many entities, that made different choices, without common management or interoperability. But large institutional data-sharing networks begin to emerge. South Korea very recently launched an initiative to build a national wide data network focused on intensive care. United States is building Chorus4ai, an analysis platform pooling data from 14 university hospitals [25]. To unlock the potential of clinical data, the German Medical Informatics Initiative [26] created 4 consortia in 2018. They aim at developing technical and organizational solutions to improve the consistency of clinical data.

Israel stands out as one of the rare countries that pooled together both claims and clinical data at a large scale: half of the population depends on 1 single healthcare provider and insurer [27].

**Fig 1:**
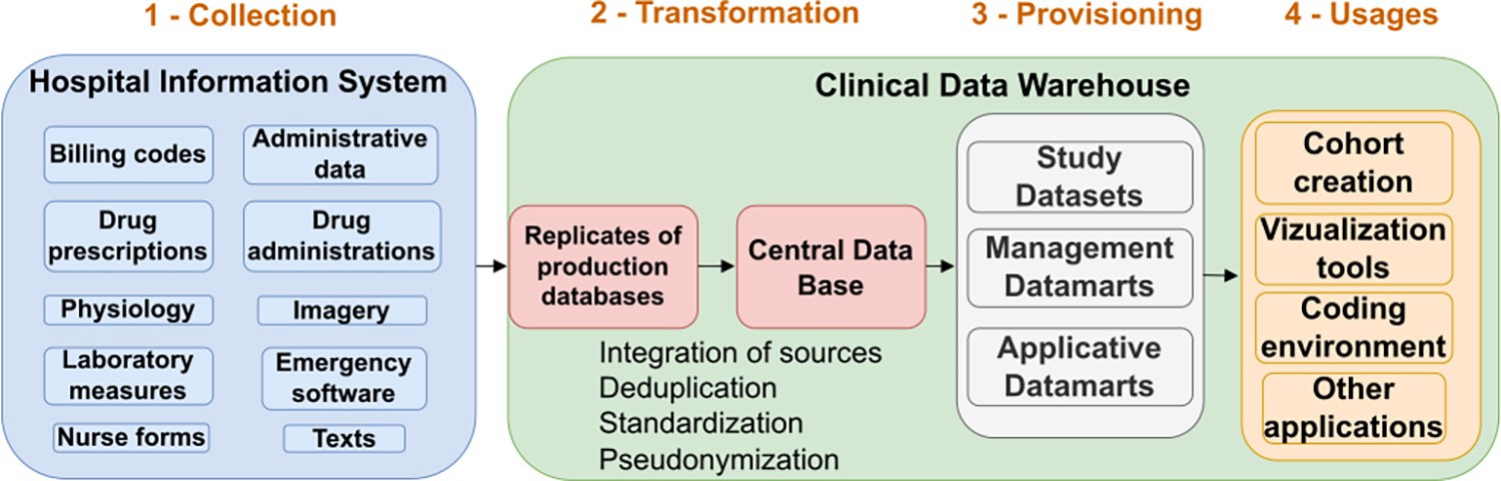
CDW: Four steps of data flow from the Hospital Information System: (1) collection, (2) transformations, and (3) provisioning. CDW, clinical data warehouse.

**An infrastructure** is needed to pool data data from 1 or more medical information systems—whatever the organizational framework—to homogeneous formats, for management, research, or care reuses [28,29]. Fig 1 illustrates for a CDW, the 4 phases of data flow from the various sources that make up the HIS:

1. **Collection** and copying of original sources.
2. **Transformation**: Integration and harmonization.
  - Integration of sources into a unique database.
  - Deduplication of identifiers.
  - Standardization: A unique data model, independent of the software models harmonizes the different sources in a common schema, possibly with common nomenclatures.
  - Pseudonymization: Removal of directly identifying elements.
3. **Provision** of subpopulation data sets and transformed datamarts for primary and secondary reuse.
4. **Usages** thanks to dedicated applications and tools accessing the datamarts and data sets.

In France, the national insurer collects all hospital activity and city care claims into a unique reimbursement database [13]. However, clinical data is historically scattered at each care site in numerous HISs. Several hospitals deployed efforts for about 10 years to create CDWs from electronic medical records [30–39]. This work has accelerated recently, with the beginning of CDWs structuring at the regional and national levels. Regional cooperation networks are being set up—such as the Ouest Data Hub [40]. In July 2022, the Ministry of Health opened a 50 million euros call for projects to set up and strengthen a network of hospital CDWs coordinated with the national platform, the Health Data Hub by 2025.

### Objective

Based on an overview of university hospital CDWs in France, this study makes general recommendations for properly leveraging the potential of CDWs to improve healthcare. It focuses on: governance, transparency, types of data, data reuse, technical tools, documentation, and data quality control processes.

## Material and methods

Interviews were conducted from March to November 2022 with 32 French regional and university hospitals, both with existing and prospective CDWs.

### Ethics statement

This work has been authorized by the board of the French High Authority of Health (HAS). Every interviewed participant was asked by email for their participation and informed on the possible forms of publication: a French official report and an international publication. Furthermore, at each interview, every participant has been asked for their agreement before recording the interview. Only 1 participant refused the video to be recorded.

### Interviews

Semi-structured interviews were conducted on the following themes: the initiation and construction of the CDWs, the current status of the project and the studies carried out, opportunities and obstacles, and quality criteria for observational research. S1 Table lists all interviewed people with their team title. The complete form, with the precised questions, is available in S2 Table.

The interview form was sent to participants in advance and then used as a support to conduct the interviews. The interviews lasted 90 min and were recorded for reference.

### Quantitative methods

Three tables detailed the structured answers in S1 Text. The first 2 tables deal with the characteristics of the actors and those of the data warehouses. We completed them based on the notes taken during the interviews, the recordings, and by asking the participants for additional information. The third table focuses on ongoing studies in the CDWs. We collected the list of these studies from the dedicated reporting portals, which we found for 8 out of 14 operational CDWs. We developed a classification of studies, based on the typology of retrospective studies described by the OHDSI research network [41]. We enriched this typology by comparing it with the collected studies resulting in the 6 following categories:

- **Outcome frequency**: Incidence or prevalence estimation for a medically well-defined target population.
- **Population characterization**: Characterization of a specific set of covariates. Feasibility and prescreening studies belong to this category [42].
- **Risk factors**: Identification of covariates most associated with a well-defined clinical target (disease course, care event). These studies look at association study without quantifying the causal effect of the factors on the outcome of interest.
- **Treatment effect**: Evaluation of the effect of a well-defined intervention on a specific outcome target. These studies intend to show a causal link between these 2 variables [43].
- **Development of diagnostic and prognostic algorithms**: Improve or automate a diagnostic or prognostic process, based on clinical data from a given patient. This can take the form of a risk, a preventive score, or the implementation of a diagnostic assistance system. These studies are part of the individualized medicine approach, with the goal of inferring relevant information at the level of individual patient’s files.
- **Medical informatics**: Methodological or tool oriented. These studies aim to improve the understanding and capacity for action of researchers and clinicians. They include the evaluation of a decision support tool, the extraction of information from unstructured data, or automatic phenotyping methods.

Studies were classified according to this nomenclature based on their title and description.

## Results

**Fig 2:**
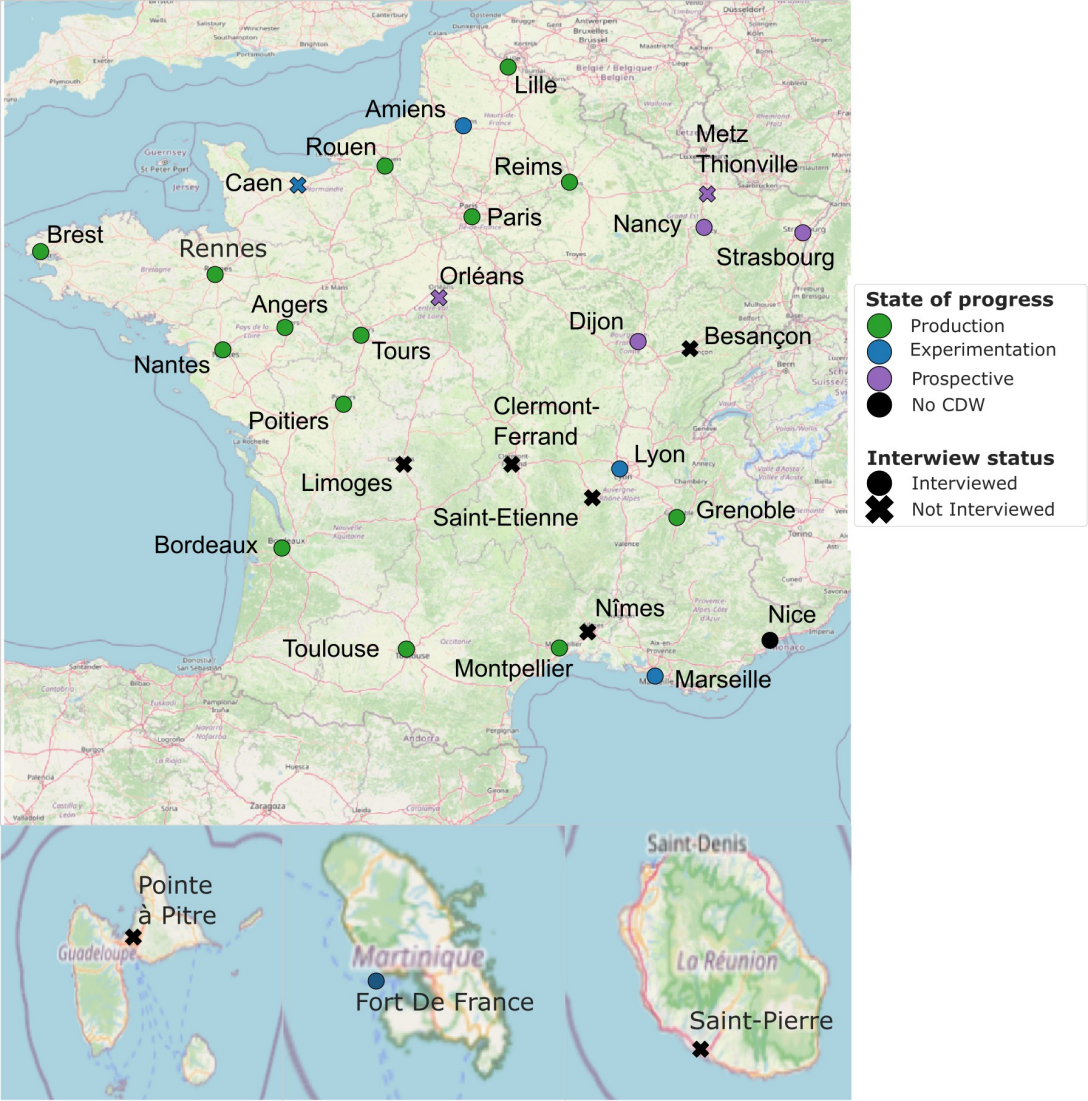
Repartition of CDWs in France. Base map and data from OpenStreetMap and OpenStreetMap Foundation. Link to the base layer of the map: https://github.com/mapnik/mapnik. CDW, clinical data warehouse.

Fig 2 summarizes the development state of progress of CDWs in France. Out of 32 regional and university hospitals in France, 14 have a CDW in production, 5 are experimenting, 5 have a prospective CDW project, 8 did not have any CDW project at the time of writing. The results are described for all projects that are at least in the prospective stage minus the 3 that we were unable to interview after multiple reminders (Orléans, Metz, and Caen), resulting in a denominator of 21 university hospitals.

### Governance

**Fig 3:**
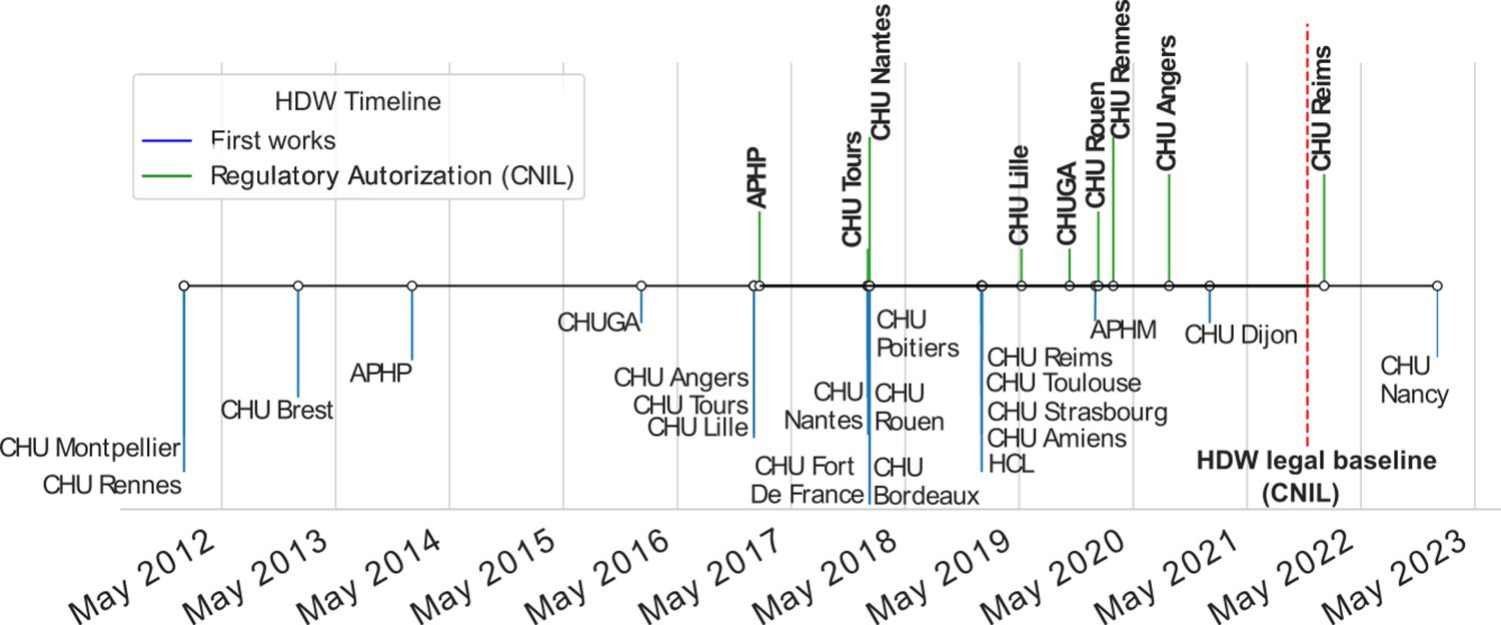
The French CDWs implementations date back to the first academic works in data reuse in early 2010s and accelerated recently. CDW, clinical data warehouse.

Fig 3 shows the history of the implementation of CDWs. A distinction must be made between the first works—in blue—, which systematically precede the regulatory authorization—in green—from the French Commission on Information Technology and Liberties (CNIL).

The CDWs have so far been initiated by 1 or 2 people from the hospital world with an academic background in bioinformatics, medical informatics, or statistics. The sustainability of the CDW is accompanied by the construction of a cooperative environment between different actors: Medical Information Department (MID), Information Systems Department (IT), Clinical Research Department (CRD), clinical users, and the support of the management or the Institutional Medical Committee. It is also accompanied by the creation of a team, or entity, dedicated to the maintenance and implementation of the CDW. More recent initiatives, such as those of the HCL (Hospitals of the city of Lyon) or the *Grand-Est* region, are distinguished by an initial, institutional, and high-level support.

The CDW has a federating potential for the different business departments of the hospital with the active participation of the CRD, the IT Department, and the MID. Although there is always an operational CDW team, the human resources allocated to it vary greatly: from half a full-time equivalent to 80 people for the AP-HP, with a median of 6.0 people. The team systematically includes a coordinating physician. It is multidisciplinary with skills in public health, medical informatics, informatics (web service, database, network, infrastructure), data engineering, and statistics.

Historically, the first CDWs were based on in-house solution development. More recently, private actors are offering their services for the implementation and implementation of CDWs (15/21). These services range from technical expertise in order to build up the data flows and data cleaning up to the delivery of a platform integrating the different stages of data processing.

### Management of studies

Before starting, projects are systematically analyzed by a scientific and ethical committee. A local submission and follow-up platform is often mentioned (12/21), but its functional scope is not well defined. It ranges from simple authorization of the project to the automatic provision of data into a Trusted Research Environment (TRE) [44]. The processes for starting a new project on the CDW are always communicated internally but rarely documented publicly (8/21).

### Transparency

Ongoing studies in CDWs are unevenly referenced publicly on hospital websites. Some institutions have comprehensive study portals, while others list only a dozen studies on their public site while mentioning several hundreds ongoing projects during interviews. In total, we found 8 of these portals out of 14 CDWs in production. Uses other than ongoing scientific studies are very rarely documented. The publication of the list of ongoing studies is very heterogeneous and fragmented between several sources: clinicaltrials.gov, the mandatory project portal of the Health Data Hub [45] or the website of the hospital data warehouse.

### Data

#### Strong dependance to the HIS

CDW data reflect the HIS used on a daily basis by hospital staff. Stakeholders point out that the quality of CDW data and the amount of work required for rapid and efficient reuse are highly dependent on the source HIS. The possibility of accessing data from an HIS in a structured and standardized format greatly simplifies its integration into the CDW and then its reuse.

#### Categories of data

Although the software landscape is varied across the country, the main functionalities of HIS are the same. We can therefore conduct an analysis of the content of the CDWs, according to the main categories of common data present in the HIS.

The common base for all CDWs is constituted by data from the Patient Administrative Management software (patient identification, hospital movements) and the billing codes. Then, data flows are progressively developed from the various softwares that make up the HIS. The goal is to build a homogeneous data schema, linking the sources together, controlled by the CDW team. The prioritization of sources is done through thematic projects, which feed the CDW construction process. These projects improve the understanding of the sources involved, by confronting the CDW team with the quality issues present in the data.

Table 1 presents the different ratio of data categories integrated in French CDWs. Structured biology and texts are almost always integrated (20/21 and 20/21). The texts contain a large amount of information. They constitute unstructured data and are therefore more difficult to use than structured tables. Other integrated sources are the hospital drug circuit (prescriptions and administration, 16/21), Intense Care Unit (ICU, 2/21), or nurse forms (4/21). Imaging is rarely integrated (4/21), notably for reasons of volume. Genomic data are well identified, but never integrated, even though they are sometimes considered important and included in the CDW work program.

**Table 1:** Type of data integrated into the French CDWs.

Category of data	Number of CDW	Ratio
Administrative	21	100%
Billing codes	20	95%
Biology	20	95%
Texts	2	95%
Drugs	16	76%
Imagery	4	19%
Nurse forms	4	19%
Anatomical pathology	3	14%
ICU	2	10%
Medical devices	2	10%

CDW, clinical data warehouse.

#### Data reuse

Today, the main use put forward for the constitution of CDWs is that of scientific research.

**Fig 4:**
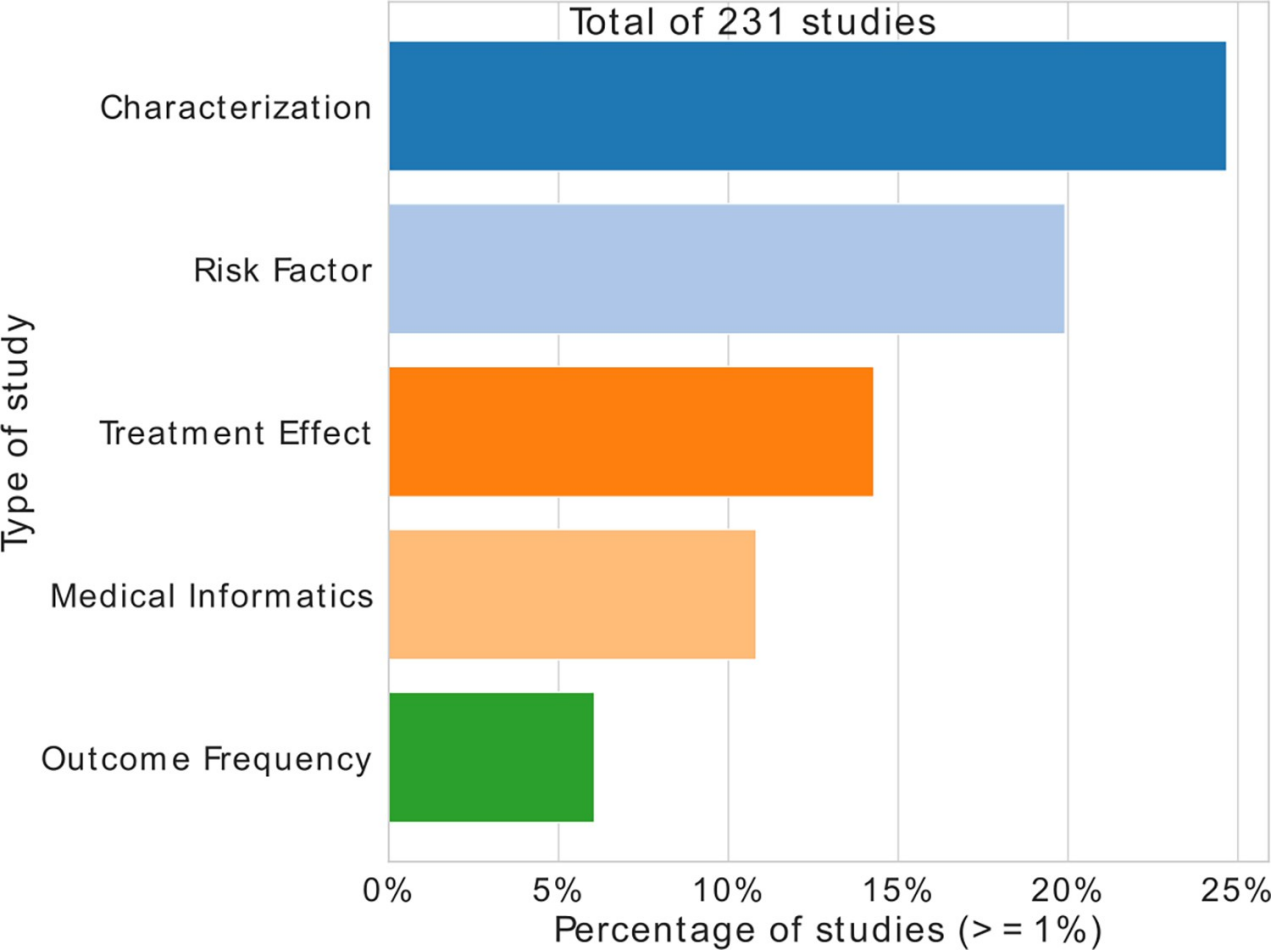
Percentage of studies by objective.

The studies are mainly observational (non-interventional). Fig 4 presents the distribution of the 6 categories defined in Quantitative methods for 231 studies collected on the study portals of 9 hospitals. The studies focus first on population characterization (25%), followed by the development of decision support processes (24%), the study of risk factors (18%), and the treatment effect evaluations (16%).

The CDWs are used extensively for internal projects such as student theses (at least in 9/21) and serve as an infrastructure for single-service research: their great interest being the de-siloing of different information systems. For most of the institutions interviewed, there is still a lack of resources and maturity of methods and tools for conducting inter-institutional research (such as in the *Grand-Ouest* region of France) or via European calls for projects (EHDEN). These 2 research networks are made possible by supra-local governance and a common data schema, respectively, eHop [46] and OMOP [47]. The Paris hospitals, thanks to its regional coverage and the choice of OMOP, is also well advanced in multicentric research. At the same time, the *Grand-Est* region is building a network of CDW based on the model of the *Grand-Ouest* region, also using eHop.

#### CDW are used for monitoring and management (16/21)

The CDW have sometimes been initiated to improve and optimize billing coding (4/21). The clinical texts gathered in the same database are queried using keywords to facilitate the structuring of information. The data are then aggregated into indicators, some of which are reported at the national level. The construction of indicators from clinical data can also be used for the administrative management of the institution. Finally, closer to the clinic, some actors state that the CDW could also be used to provide regular and appropriate feedback to healthcare professionals on their practices. This feedback would help to increase the involvement and interest of healthcare professionals in CDW projects. The CDW is sometimes of interest for health monitoring (e.g., during COVID-19) or pharmacovigilance (13/21).

#### Strong interest for CDW in the context of care (13/21)

Some CDWs develop specific applications that provide new functionalities compared to care software. Search engines can be used to query all the hospital’s data gathered in the CDW, without data compartmentalization between different softwares. Dedicated interfaces can then offer a unified view of the history of a patient’s data, with inter-specialty transversality, which is particularly valuable in internal medicine. These cross-disciplinary search tools also enable healthcare professionals to conduct rapid searches in all the texts, for example, to find similar patients [32]. Uses for prevention, automation of repetitive tasks, and care coordination are also highlighted. Concrete examples are the automatic sorting of hospital prescriptions by order of complexity or the setting up of specialized channels for primary or secondary prevention.

### Technical architecture

The technical architecture of modern CDWs has several layers:

- Data processing: connection and export of source data, diverse transformation (cleaning, aggregation, filtering, standardization).
- Data storage: database engines, file storage (on file servers or object storage), indexing engines to optimize certain queries.
- Data exposure: raw data, APIs, dashboards, development and analysis environments, specific web applications.

Supplementary cross-functional components ensure the efficient and secure operation of the platform: identity and authorization management, activity logging, automated administration of servers and applications.

The analysis environment (Jupyterhub or RStudio datalabs) is a key component of the platform, as it allows data to be processed within the CDW infrastructure. A few CDWs had such operational datalab at the time of our study (6/21) and almost all of them have decided to provide it to researchers. Currently, clinical research teams are still often working on data extractions in less secure environments.

### Data quality, standard formats

#### Quality tools

Systematic data quality monitoring processes are being built in some CDWs. Often (8/21), scripts are run at regular intervals to detect technical anomalies in data flows. Rare data quality investigation tools, in the form of dashboards, are beginning to be developed internally (3/21). Theoretical reflections are underway on the possibility of automating data consistency checks, for example, demographic or temporal. Some facilities randomly pull records from the EHR to compare them with the information in the CDW.

#### Standard format

No single standard data model stands out as being used by all CDWs. All are aware of the existence of the OMOP (research standard) [47] and HL7 FHIR (communication standard) models [48]. Several CDWs consider the OMOP model to be a central part of the warehouse, particularly for research purposes (9/21). This tendency has been encouraged by the European call for projects EHDEN, launched by the OHDSI research consortium, the originator of this data model. In the *Grand-Ouest* region of France, the CDWs use the eHop warehouse software. The latter uses a common data model also named eHop. This model will be extended with the future warehouse network of the *Grand Est* region also choosing this solution. Including this grouping and the other establishments that have chosen eHop, this model includes 12 establishments out of the 32 university hospitals. This allows eHop adopters to launch ambitious interregional projects. However, eHop does not define a standard nomenclature to be used in its model and is not aligned with emerging international standards.

#### Documentation

Half of the CDWs have put in place documentation accessible within the organization on data flows, the meaning and proper use of qualified data (10/21 mentioned). This documentation is used by the team that develops and maintains the warehouse. It is also used by users to understand the transformations performed on the data. However, it is never publicly available. No schema of the data once it has been transformed and prepared for analysis is published.

## Discussion

### Principal findings

We give the first overview of the CDWs in university hospitals of France with 32 hospitals reviewed. The implementation of CDW dates from 2011 and accelerated in the late 2020. Today, 24 of the university hospitals have an ongoing CDW project. From this case study, some general considerations can be drawn that should be valuable to all healthcare system implementing CDWs on a national scale.

### Governance

As the CDW becomes an essential component of data management in the hospital, the creation of an autonomous internal team dedicated to data architecture, process automation, and data documentation should be encouraged [44]. This multidisciplinary team should develop an excellent knowledge of the data collection process and potential reuses in order to qualify the different flows coming from the source IS, standardize them towards a homogenous schema and harmonize the semantics. It should have a sound knowledge of public health, as well as the technical and statistical skills to develop high-quality software that facilitates data reuse.

The resources specific to the warehouse are rare and often taken from other budgets or from project-based credits. While this is natural for an initial prototyping phase, it does not seem adapted to the perennial and transversal nature of the tool. As a research infrastructure of growing importance, it must have the financial and organizational means to plan for the long term.

The governance of the CDW has multiple layers: local within the university hospital, interregional, and national/international. The first level allow to ensure the quality of data integration as well as the pertinence of data reuse by clinicians themselves. The interregional level is well adapted for resources mutualization and collaboration. Finally, the national and international levels assure coordination, encourage consensus for committing choices such as metadata or interoperability, and provide financial, technical, and regulatory support.

### Transparency

Health technology assessment agencies advocate for public registration of comparative observational study protocols before conducting the analysis [8,17,49]. They often refer to clinicaltrials.gov as potential but not ideal registration portal for observational studies. The research community advocates for public registrations of all observational studies [50,51]. More recently, it emphasizes the need for more easy data access and the publication of study code [29,52,53]. We embrace these recommendations and we point to the unfortunate duplication of these study reporting systems in France. One source could be favored at the national level and the second one automatically fed from the reference source, by agreeing on common metadata.

From a patient’s perspective, there is currently no way to know if their personal data is included for a specific project. Better patient information about the reuse of their data is needed to build trust over the long term. A strict minimum is the establishment and update of the declarative portals of ongoing studies at each institution.

### Data and data usage

When using CDW, the analyst has not defined the data collection process and is generally unaware of the context in which the information is logged. This new dimension of medical research requires a much greater development of data science skills to change the focus from the implementation of the statistical design to the data engineering process. Data reuse requires more effort to prepare the data and document the transformations performed.

The more heterogeneous a HIS system is, the less qualitative would be the CDW built on top of it. There is a need for increasing interoperability, to help EHR vendors interfacing the different hospital softwares, thus facilitating CDW development. One step in this direction would be the open source publication of HIS data schema and vocabularies. At the analysis level, international recommendations insist on the need for common data formats [52,54]. However, there is still a lack of adoption of research standards from hospital CDWs to conduct robust studies across multiple sites. Building open-source tools on top of these standards such as those of OHDSI [41] could foster their adoption. Finally, in many clinical domains, sufficient sample size is hard to obtain without international data-sharing collaborations. Thus, more incitation is needed to maintain and update the terminology mappings between local nomenclatures and international standards.

Many ongoing studies concern the development of decision support processes whose goal is to save time for healthcare professionals. These are often research projects, not yet integrated into routine care. The analysis of study portals and the interviews revealed that data reuse oriented towards primary care is still rare and rarely supported by appropriate funding. The translation from research to clinical practice takes time and need to be supported on the long run to yield substantial results.

### Technical architecture

Tools, methods, and data formats of CDW lack harmonization due to the strong technical innovation and the presence of many actors. As suggested by the recent report on the use of data for research in the UK [44], it would be wise to focus on a small number of model technical platforms.

These platforms should favor open-source solutions to assure transparency by default, foster collaboration and consensus, and avoid technological lock-in of the hospitals.

### Data quality and documentation

Quality is not sufficiently considered as a relevant scientific topic itself. However, it is the backbone of all research done within a CDW. In order to improve the quality of the data with respect to research uses, it is necessary to conduct continuous studies dedicated to this topic [52,54–56]. These studies should contribute to a reflection on methodologies and standard tools for data quality, such as those developed by the OHDSI research network [41].

Finally, there is a need for open-source publication of research code to ensure quality retrospective research [55,57]. Recent research in data analysis has shown that innumerable biases can lurk in training data sets [58,59]. Open publication of data schemas is considered an indispensable prerequisite for all data science and artificial intelligence uses [58]. Inspired by data set cards [58] and data set publication guides, it would be interesting to define a standard CDW card documenting the main data flows.

### Limitations

The interviews were conducted in a semi-structured manner within a limited time frame. As a result, some topics were covered more quickly and only those explicitly mentioned by the participants could be recorded. The uneven existence of study portals introduces a bias in the recording of the types of studies conducted on CDW. Those with a transparency portal already have more maturity in use cases.

For clarity, our results are focused on the perimeter of university hospitals. We have not covered the exhaustive healthcare landscape in France. CDW initiatives also exist in primary care, in smaller hospital groups and in private companies.

## Conclusions

The French CDW ecosystem is beginning to take shape, benefiting from an acceleration thanks to national funding, the multiplication of industrial players specializing in health data and the beginning of a supra-national reflection on the European Health Data Space [60]. However, some points require special attention to ensure that the potential of the CDW translates into patient benefits.

The priority is the creation and perpetuation of multidisciplinary warehouse teams capable of operating the CDW and supporting the various projects. A combination of public health, data engineering, data stewardship, statistics, and IT competences is a prerequisite for the success of the CDW. The team should be the privileged point of contact for data exploitation issues and should collaborate closely with the existing hospital departments.

The constitution of a multilevel collaboration network is another priority. The local level is essential to structure the data and understand its possible uses. Interregional, national, and international coordination would make it possible to create thematic working groups in order to stimulate a dynamic of cooperation and mutualization.

A common data model should be encouraged, with precise metadata allowing to map the integrated data, in order to qualify the uses to be developed today from the CDWs. More broadly, open-source documentation of data flows and transformations performed for quality enhancement would require more incentives to unleash the potential for innovation for all health data reusers.

Finally, the question of expanding the scope of the data beyond the purely hospital domain must be asked. Many risk factors and patient follow-up data are missing from the CDWs, but are crucial for understanding pathologies. Combining city data and hospital data would provide a complete view of patient care.

## Supporting information

S1 Table List of interviewed stakeholders with their teams. (XLSX)

S2 Table Interview form. (XLSX)

S1 Text Study data tables. (DOCX)
