# Correction: Good practices for clinical data warehouse implementation: A case study in France

**DOI:** 10.1371/journal.pdig.0000369

**Published:** 2023-09-29

**Authors:** Matthieu Doutreligne, Adeline Degremont, Pierre-Alain Jachiet, Antoine Lamer, Xavier Tannier

Fig 4 is incorrect. The authors have provided a corrected version here.

**Fig 4:**
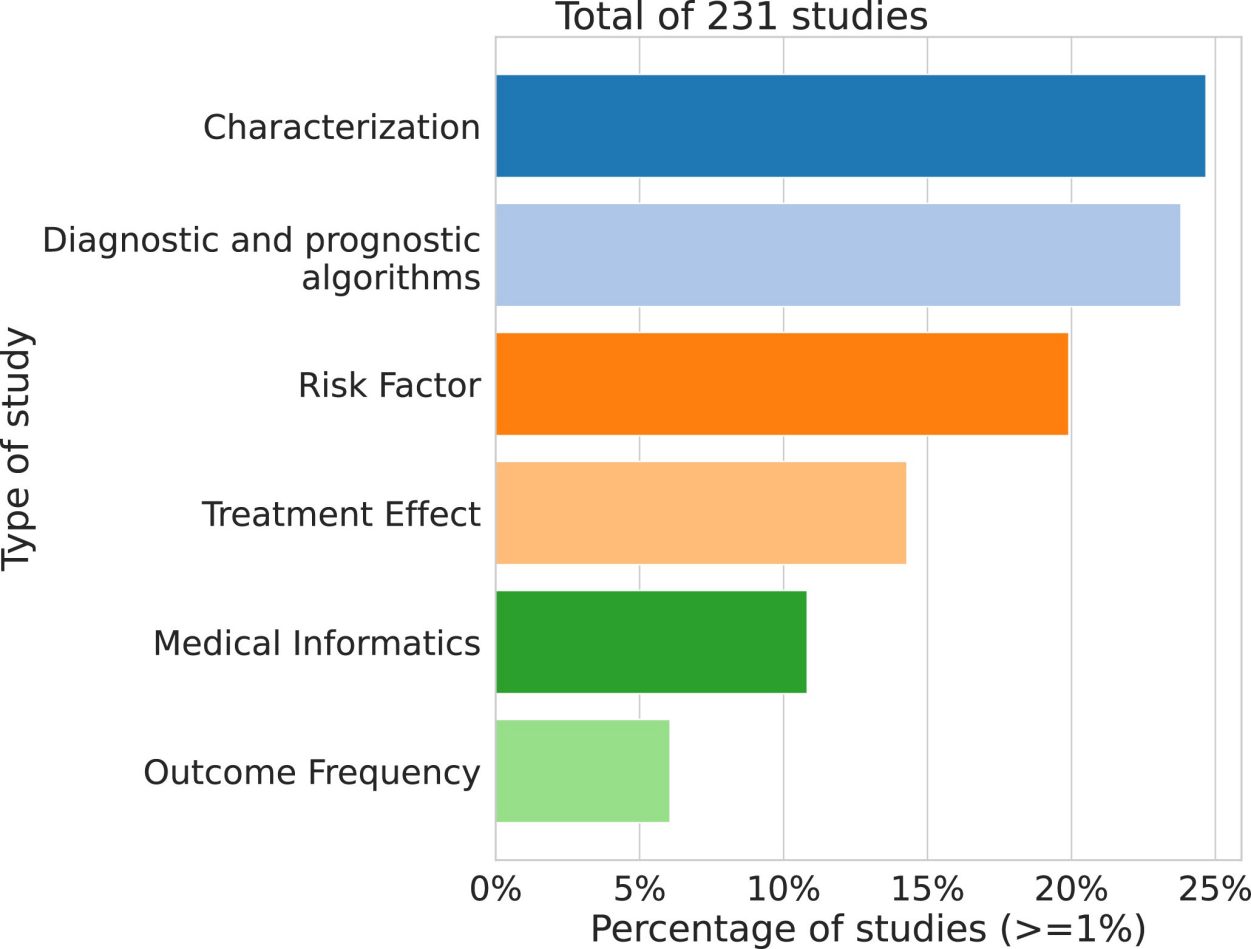
Percentage of studies by objective. https://doi.org/10.1371/journal.pdig.0000298.g004.
