# Supplementary material for: Good practices for clinical data warehouse implementation: A case study in France
Source: PLOS Digit Health. 2023 Jul 6;2(7):e0000298. doi: 10.1371/journal.pdig.0000298 (PMC10325086; doi:10.1371/journal.pdig.0000298)
Supplement: S1 Text — (DOCX) [file pdig.0000298.s003.docx]

The data tables used to produce the figures in the results section are available at the following url: <https://gitlab.has-sante.fr/has-sante/public/rapport_edsh/> .

The guests table concerns the individuals interviewed, the interview dates, the positions and the membership of a specific team. The warehouse table collects information about the CDW. The table of studies is the referencing of the studies informed on 10 portals of studies in progress (or completed if available) available in free access.
